# Different dietary assessment methods, similar conclusions? Comparison of a country’s adherence to food-based dietary guidelines as depicted in two population-based surveys using different dietary assessment methods

**DOI:** 10.1017/S1368980022000647

**Published:** 2022-09

**Authors:** Nena Karavasiloglou, Giulia Pestoni, Anna Dehler, Janice Sych, David Faeh, Sabine Rohrmann

**Affiliations:** 1 Division of Chronic Disease Epidemiology, Epidemiology, Biostatistics and Prevention Institute, University of Zurich, Hirschengraben 84, Zurich CH-8001, Switzerland; 2 Nutrition Group, Health Department, Swiss Distance University of Applied Sciences, Zurich, Switzerland; 3 Institute of Food and Beverage Innovation, ZHAW Zurich University of Applied Sciences, Wädenswil, Switzerland; 4 Health Department, Bern University of Applied Sciences, Bern, Switzerland

**Keywords:** Food-based guidelines, Diet, Assessment, Survey, Population-based

## Abstract

**Objective:**

Different methods of dietary intake assessment are frequently used to assess a population’s diet. In this study, we aimed to compare the adherence to Swiss food-based dietary guidelines as depicted in two Swiss population-based surveys using different methods of dietary assessment.

**Design:**

Two population-based, cross-sectional surveys were compared. In the Swiss Health Survey (SHS), diet was assessed via a short set of questions on specific food groups, while in menuCH by two non-consecutive 24-h dietary recall interviews.

**Setting:**

To compare the diet depicted in these surveys, we used the Swiss food-based dietary guidelines on vegetable, fruit, dairy product, meat and meat product, fish and alcohol. The weighted proportion of responders meeting these guidelines was calculated for both surveys and was compared overall and by selected characteristics.

**Participants:**

Residents of Switzerland, selected from a stratified random sample of the non-institutionalised residents, who agreed to participate in the respective survey. To ensure comparability between the surveys, the age of the study populations was restricted to 18–75 years.

**Results:**

In menuCH, approximately 2 % of responders met ≥4 of the selected Swiss food-based dietary guidelines. In the SHS, using a cruder dietary assessment, the corresponding percentage was 20 %. In both surveys, more women and never smokers were meeting ≥4 food-based dietary guidelines compared to men and current or former smokers, respectively.

**Conclusions:**

Our study comparing the diet in two population-based, representative surveys detected large variations in guideline adherence depending on the dietary assessment method used.

The consistent associations between diet and disease morbidity and mortality from many non-communicable diseases^(1,2)^ in the scientific literature have highlighted the importance of healthy diet in disease prevention. Many professional organisations and scientific societies have published food-based dietary guidelines to help individuals make healthier nutrition choices. However, following a high-quality diet is not equally easy for all, with specific population groups reporting lower diet quality than others^(3)^. Thus, many countries and scientific institutions have aimed to monitor the adherence to dietary recommendations^(4)^ to assess current dietary habits and pinpoint areas or population subgroups where actions are needed.

The accurate assessment of diet at population level is challenging. Different assessment methods of dietary intake have been used in studies and various validated dietary assessment tools have been developed to aid researchers in estimating individual dietary intake. In large-scale population-based studies, including national nutrition surveys, the most commonly used dietary assessment methods are 24-h dietary recall(s), FFQ and brief screeners of dietary intake. While the advantages and disadvantages of these methods have been described^(4,5)^, a universally agreed-upon approach is still lacking in studies assessing diet at the population level. Given the different methods of dietary assessment available, studies and nutrition surveys should aim to increase the level of precision of dietary intake measures while minimising the burden of the participants.

In this study, we aimed to compare the adherence to the Swiss food-based dietary guidelines as depicted in two surveys representative of Switzerland that made use of different dietary assessment methods. The adherence to the Swiss food-based dietary guidelines was explored overall and by selected socio-demographic and lifestyle characteristics.

## Methods

### Data and study populations

#### Swiss Health Survey

The Swiss Health Survey (SHS) is a population-based, cross-sectional, nationwide survey conducted every 5 years since 1992. It collects information on the health status, lifestyle, diet and demographic factors of the population living in Switzerland. A stratified random sampling technique based on inhabitants’ registries is used, including individuals 15 years or older who live in a private household. A computer-assisted telephone interview followed by a written questionnaire (paper or online) is provided upon approval from the participants^(6)^.

In our study, we used data of the SHS conducted in 2012 that included 21 597 individuals. The survey was conducted in the three main languages of Switzerland (German, French and Italian) and was carried out throughout the year to account for seasonal variation. Participation rate was 53 %. To ensure comparability between the study populations, we included in the analyses SHS responders aged 18–75 years. This led to a final analytical sample of 18 991 SHS responders.

In the SHS, information on dietary intake was collected via a short set of questions on selected food groups. These questions were not part of a FFQ, but of an extensive questionnaire assessing different health behaviours of the population in Switzerland. This questionnaire is not validated for dietary intake. For fruits, vegetables and dairy products, responders were asked the habitual frequency of consumption of the overall food groups (in d/week), and if it exceeded four times a week, the quantity of consumption (in portions/d). In responders with frequency of consumption higher than four times a week, but missing quantity of consumption, the latter was replaced with the sex- and age-specific median quantity of consumption (in portions/d) for each frequency of consumption and food group. For meat, meat products and fish, responders were only asked the frequency of consumption of the overall food groups (in d/week). For these, we assumed a quantity of consumption of one portion per day. Finally, frequency and quantity of alcohol intake were assessed, but the questions on quantity of consumption only covered the past 7 d and not the usual consumption, as for the other food groups. For this reason, we decided to only consider the frequency of consumption (in d/week) in this study and assumed again a quantity of consumption of one portion per day, unless the reported frequency was multiple times per day.

#### National nutrition survey menuCH

menuCH is a population-based, cross-sectional survey that was conducted in Switzerland in 2014 and 2015. The study population consisted of a stratified random sample of non-institutionalised residents aged 18–75 years that was provided by the Federal Statistical Office. The survey was designed to be representative of the major areas of Switzerland (Eurostat NUTS-2), the three main linguistic regions and predefined age categories (18–29, 30–44, 45–59 and 60–75 years). Participation rate was 38 %. A complete flow diagram of study participation was previously published^(7)^.

Dietary intake was assessed by two non-consecutive 24-h dietary recall interviews^(8)^. Briefly, the first 24-h dietary recall interview took place in person and the second over the phone, approximately 2–6 weeks after the first, equally distributed across all weekdays and seasons. To aid in the quantification of the amount of food consumed, a book with 119 series of six graduated portion size pictures and a set of about 60 household measures were used^(9)^. Standard recipes were classified by trained dietitians, and they contribute in the food groups assessed. Food consumption of participants was recorded using the trilingual Swiss version (0.2014.02.27) of the software GloboDiet® (formerly EPIC-Soft®, International Agency for Research on Cancer IARC, Lyon, France^(10,11)^, adapted for Switzerland by the Federal Food Safety and Veterinary Office, Bern, Switzerland). Data cleaning was done after the completion of data collection using an updated version of the GloboDiet® software (0.2015.09.28).

### Socio-demographic and lifestyle variables

In both surveys, information on socio-demographic and lifestyle variables was collected through questionnaires^(6,7)^. To allow for comparison between the surveys, the variables were categorised identically.

In the SHS, body weight and height were self-reported^(6)^. In menuCH, body weight and height were measured following standard procedures^(8)^, and for pregnant and lactating women or when measuring was not possible, the self-reported values for body weight (*n* 34) and height (*n* 7) were used. For pregnant women, the self-reported weight reflected their pre-gestational weight. In both surveys, BMI was calculated and categorised according to guidelines of the World Health Organization^(12)^.

### Swiss food-based dietary guidelines and their operationalisation in the datasets

The Swiss Society for Nutrition, together with the Federal Food Safety and Veterinary Office, issued the updated Swiss Food Pyramid in 2011^(13)^. In it, food groups are depicted in six levels of consumption. Some food groups are accompanied by detailed recommendations on consumption frequency (e.g. portions/d or week), whereas others are given without an exact suggested consumption frequency. To allow for comparison between the two study populations, the present study used only the food-based dietary guidelines that could be operationalised in both surveys.

Detailed information on the operationalisation of the food-based dietary guidelines for each survey can be found in Supplemental Table 1. Briefly, we were able to operationalise the food-based dietary guidelines on vegetable, fruit, dairy product, meat and meat product and fish consumption, as well as the one on alcohol intake. Since the recommendation on alcohol intake is not specific, we based our classification on the Commission For Alcohol, which recommended people to include alcohol-free days during the week^(14)^, and on previous studies conducted in Switzerland, which set the limit to 15 g/d for women and 30 g/d for men^(7)^. To be able to account for both, we set the recommendation as 1–2 times/week alcohol consumption in the SHS and a habitual intake of 7·5 g for women and 15 g for men in menuCH. The number of guidelines met was summed for each participant and further categorised as <2, 2–3 or ≥4 guidelines.

### Statistical analyses

Baseline characteristics of study participants were reported as percentages for both surveys.

In menuCH, habitual dietary intake was estimated based on the two non-consecutive 24-h dietary recall interviews using the Multiple Source Method^(15)^. Participants reporting avoidance of specific food groups in the menuCH questionnaire and reporting zero consumption in both 24-h dietary recall interviews were considered as never-consumers for the models estimating habitual intake. Sensitivity analyses were conducted with sex and age as adjusting factors in the Multiple Source Method models.

In both surveys, the operationalisation of the food-based dietary guidelines was reported as weighted proportions of study participants meeting compared to not meeting the food-based dietary guidelines. Survey-specific weights were used to account for design and non-response. In both, the SHS and menuCH, the survey weights were based on age, sex, marital status, major area, nationality and household size. In menuCH, food consumption was further weighted for the uneven distribution of the two 24-h dietary recall interviews over seasons and weekdays.

The analyses were conducted using R software (version 4.1.1. for Windows, R Foundation for Statistical Computing) and the web-based application of the Multiple Source Method programme^(16)^. The package *survey* ^(17)^ was used to perform the weighted statistical analyses.

## Results

In both surveys, responders were balanced between sexes and had a similar distribution of participants in terms of linguistic regions, age categories, BMI categories and self-reported health (Table 1). Differences were observed between the two study populations in education and smoking status, with menuCH responders having completed tertiary education more frequently and identifying less frequently as current smokers. When looking at the Swiss food-based dietary guidelines, two different pictures emerged. In the SHS, the majority of responders met 2–3 of the operationalised food-based dietary guidelines, whereas more than half of the menuCH responders met <2 of the operationalised food-based dietary guidelines.

**Table 1:** Characteristics of the Swiss Health Survey and the menuCH participants

	Swiss Health Survey	menuCH*
	%	%
Count, unweighted		
* n*	18 991	2057
Sex		
* *Females	50·0	50·2
* *Males	50·0	49·8
Linguistic region		
* *German-speaking	71·3	69·2
* *French-speaking	24·2	25·5
* *Italian-speaking	4·5	5·6
Age categories		
* *18–29 years	19·8	18·8
* *30–44 years	28·3	29·9
* *45–59 years	30·6	29·8
* *60–75 years	21·3	21·6
BMI categories		
* *Underweight (BMI < 18·5 kg/m^2^)	3·3	2·4
* *Normal weight (18·5 ≤ BMI < 25·0 kg/m^2^)	54·7	54·1
* *Overweight (25·0 ≤ BMI < 30·0 kg/m^2^)	30·9	30·6
* *Obese (BMI ≥ 30·0 kg/m^2^)	10·3	12·9
* *Missing	0·8	–
Education, highest degree		
* *Primary/no degree	14·1	4·7
* *Secondary	54·4	42·6
* *Tertiary	31·0	52·6
* *Missing	0·5	0·1
Smoking status		
* *Never	48·3	42·9
* *Former	21·4	33·6
* *Current	30·3	23·3
* *Missing	0·1	0·2
Self-reported health		
* *Very bad to medium	15·4	12·7
* *Good to very good	84·5	87·1
* *Missing	0·1	0·2
Currently on a special diet (Swiss Health Survey) or weight loss diet (menuCH)		
* *Yes	9·4	5·4
* *No	90·6	94·4
* *Missing	–	0·2
Recommendations met		
* *Less than two	20·6	51·8
* *Two to three	59·8	45·9
* *Four or more	19·6	2·3

* In both surveys, survey weights were used to account for sample design and non-response based on six socio-demographic parameters (i.e. age, sex, marital status, major area, nationality and household size). In menuCH, the recommendations met were further weighted for seasons and weekdays.

BMI was based on self-reported information in the Swiss Health Survey and on measured or self-reported (when measurement was not possible) information in menuCH.

Taking sampling weights into account, a higher proportion of women met ≥4 of the food-based dietary guidelines compared to men independent of the survey (Table 2). A similar pattern was also seen for never smokers compared to former or current smokers, with the differences being more pronounced in the SHS. When examining the results by linguistic regions, we observed differences between the surveys. In the SHS, a higher proportion met ≥4 food-based dietary guidelines in the Italian-speaking region, whereas in menuCH no substantial differences across linguistic regions were observed, with only a slightly lower proportion meeting ≥4 of the guidelines in the Italian-speaking region.

**Table 2:** Weighted proportions of participants meeting the Swiss food-based dietary guidelines

	Swiss Health Survey			menuCH†		
	Less than two	Two to three	Four or more	Less than two	Two to three	Four or more
	%	%	%	%	%	%
Sex						
* *Females	12·6	60·0	27·4	50·0	46·4	3·6
* *Males	28·6	59·6	11·8	53·6	45·4	1·0
Linguistic region						
* *German-speaking	22·6	59·5	17·9	51·7	45·9	2·4
* *French-speaking	16·3	60·1	23·6	52·0	45·7	2·4
* *Italian-speaking	11·8	62·2	25·9*	52·0	46·6	1·4
Age categories						
* *18–29 years	19·8	62·7	17·5	57·7	41·0	1·4
* *30–44 years	18·7	61·2	20·1	50·7	46·6	2·7
* *45–59 years	21·4	58·8	19·8	51·6	46·0	2·5
* *60–75 years	22·7	56·6	20·7	48·5	49·2	2·4
Smoking status						
* *Never	16·9	60·3	22·9	44·9	52·1	3·1
* *Former	21·3	58·2	20·6	52·2	46·0	1·8
* *Current	26·1	60·1	13·9	63·9	34·4	1·7
* *Missing	49·4	50·6	–	63·5	36·5	–
BMI categories						
* *Underweight (BMI < 18·5 kg/m^2^)	13·3	60·8	25·9	46·2	50·4	3·4
* *Normal weight (18·5 ≤ BMI < 25·0 kg/m^2^)	19·8	59·2	21·0	48·1	49·0	2·9
* *Overweight (25·0 ≤ BMI < 30·0 kg/m^2^)	23·7	60·0	16·2	57·2	41·5	1·2
* *Obese (BMI ≥ 30·0 kg/m^2^)	18·1	62·2	19·7	55·4	42·3	2·3
* *Missing	19·3	51·8	28·9	–	–	–

* 0.1 % has missing information on the number of guidelines met.

† In both surveys, survey weights were used to account for sample design and non-response based on six socio-demographic parameters (i.e. age, sex, marital status, major area, nationality and household size). In menuCH, results were further weighted for seasons and weekdays.

**Fig. 1:**
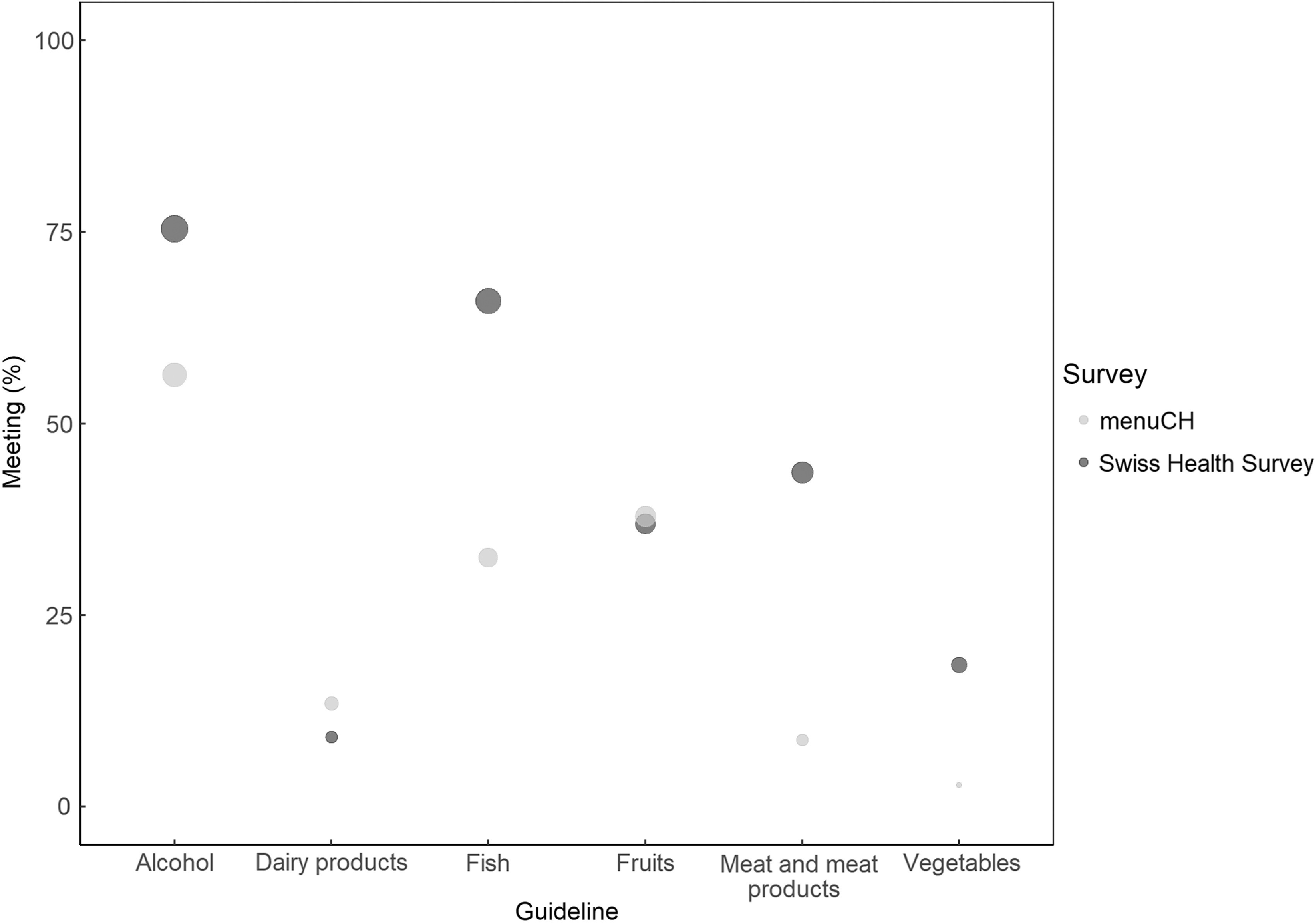
Weighted proportions of participants meeting the selected Swiss food-based dietary guidelines. The size of the circles corresponds to the weighted proportions of participants meeting each guideline. In both surveys, survey weights were used to account for sample design and non-response based on six socio-demographic parameters (i.e. age, sex, marital status, major area, nationality and household size). In menuCH, results were further weighted for seasons and weekdays

The weighted proportions of responders meeting individual food-based dietary guidelines are shown in Fig. 1. Overall, responders of the SHS met the food-based dietary guidelines at a higher proportion than menuCH responders, except for the dairy product and fruit guidelines. In the SHS, the least frequently met guideline was the one on dairy product consumption, whereas in menuCH the vegetable guideline was less frequently met. The largest differences between surveys were seen for the meat and meat product guideline (meeting the guideline: SHS 43·6 %, menuCH 8·7 %), followed by the fish guideline (meeting the guideline: SHS 66·0 %, menuCH 32·5 %). When looking at these proportions without accounting for weights, the results are largely similar (see online Supplemental Table 2). Sensitivity analyses accounting for sex and age in the estimation of habitual intake in menuCH using the Multiple Source Method revealed similar results to the ones presented as main analyses (see online Supplemental Table 3).

## Discussion

In our study comparing the diet depicted in two population-based, representative surveys we saw large differences depending on the dietary assessment method used. In the menuCH survey, which assessed diet via 24-h dietary recall interviews, 51·8 % of responders met <2 of the selected food-based dietary guidelines and 2·3 % met ≥4 of the selected food-based dietary guidelines. The results were strikingly different in the SHS, where 59·8 % met 2–3 of the selected food-based dietary guidelines and 19·6 % met ≥4 of the selected food-based dietary guidelines.

The difference found between the two population-based, representative surveys that were conducted around the same time can potentially be attributed to the dietary assessment method used. While the menuCH survey used repeated 24-h dietary recall interviews, the dietary assessment in the SHS was conducted using a small number of questions on selected food groups as part of an extensive health questionnaire. While leading organisations (e.g. European Food Safety Authority) have highlighted the use of dietary recalls as the most suitable assessment method when investigating the dietary intake at population level, the use of simpler tools like short screeners and questionnaires is still prevalent in surveys. Additionally, the reference software GloboDiet®, used in the menuCH dietary assessment, has been proven to provide reliable estimates of the consumed nutrients and foods^(10,11)^. Thus, it is likely that the diet depicted in menuCH resembles more closely the real dietary habits of people living in Switzerland.

Additionally, the slight differences between the two study populations with respect to some socio-demographic and lifestyle variables (e.g. smoking status, education) could also have contributed to the differences seen between the two surveys. However, the lower proportion of current smokers and higher number of respondents with tertiary education in menuCH probably resulted in a more favourable dietary picture with a higher proportion of respondents meeting the guidelines. If menuCH and the SHS had identical study population structures with respect to socio-demographic and lifestyle, we would expect a lower proportion of respondents meeting the guidelines in menuCH, making the differences between the two surveys even more pronounced.

Our results are largely comparable to those in the literature. Existing studies from Switzerland reported that 23–41 % of the population adhered to ≥3 of the Swiss food-based dietary guidelines^(7,18,19)^. In our study, these proportions ranged from 15·8 % (menuCH) to 48·3 % (SHS; data not shown). However, the results of these studies cannot be compared directly, due to the different number of the Swiss food-based dietary guidelines considered (seven^(7)^; six^(20)^; five^(18,19)^) and the way these were operationalised in each study, as well as the varying methods of dietary assessment used. A recent meta-analysis reported that almost 40 % of the population in both high and low/middle-income countries do not adhere to their national food-based dietary guidelines^(21)^.

When looking at the difference in proportions of meeting the food-based dietary guidelines across socio-demographic and lifestyle characteristics, we observed that women and never smokers adhered to the guidelines at a higher proportion. Studies in the literature have also reported on the higher diet quality of these population subgroups^(3,18,20)^. Regarding the differences by linguistic region, a higher proportion of individuals from the German-speaking region of Switzerland meeting more guidelines was also reported in a previous menuCH analysis^(7)^.

Despite the striking difference in the proportion of people meeting the food-based dietary guidelines as seen in both surveys, both highlight, albeit to a different extent, the low proportion of people in Switzerland meeting the food-based dietary guidelines. The low proportion of people meeting most healthy eating guidelines of the Swiss Food Pyramid has important public health implications, as it highlights the areas of the diet where more focus is needed to facilitate the adoption of healthier habits. The low adherence to the food-based dietary guidelines could be attributed to lack of awareness of the dietary guidelines, limited knowledge on how to follow the current guidelines (e.g. healthy recipes, substitution of foods) and/or lack of access to healthy foods. A previous study, examining the barriers to healthy eating in Switzerland, identified several, with taste and price most prevalently reported as perceived barriers to healthy eating^(22)^. Taste, daily habits, time and lack of willpower have also been associated with lower adherence to the Swiss food-based dietary guidelines^(20)^. Actions should be taken to raise awareness of the existence of national guidelines, remove barriers to healthy eating and make healthier foods the easiest and most readily accessible option to aid the population in adhering to the food-based dietary guidelines.

Dietary guidelines from around the western world uniformly point to the same healthy choices of consuming fruit, vegetables, fish and whole grain while limiting the amount of meat, salt and sugar in the diet. The existing literature indicates that following such healthy eating recommendations is associated with better mortality outcomes^(23,24)^. Among the different guidelines, adherence to the fruit and vegetable recommendation was seen to have the largest impact on mortality^(24)^. Overall, a large body of evidence suggests that daily adequate consumption of fruits and vegetables has a protective effect on mortality^(25–27)^, as well as death from the most common non-communicable diseases^(28)^. These results are underlining once again the importance of diet in the prevention of premature death. The vast majority of participants in both our study populations did not meet the fruit or vegetable recommendations, highlighting an area where progress is needed in terms of information, education and policy (e.g. subsidies) in order to increase fruit and vegetable consumption.

In addition to the adoption of healthier dietary habits due to their link with health outcomes, there is an urgent need to steer the population towards a more sustainable diet. Revising food pyramids and food-based dietary guidelines to consider these new priorities might attract the interest of certain population subgroups, leading to improved adherence. In fact, many organisations and nutrition societies are discussing or already issuing dietary recommendations which promote sustainability (e.g. Denmark^(29)^). Since such guidelines are generally adopting a strong position towards limiting meat and meat product consumption, considerable public health efforts will be needed to shift the consumption in Switzerland away from meat and towards more planetary healthy food options. Other considerations that already influence national food-based dietary guidelines such as the level of processing^(30)^ or socio-cultural aspects of food should also shape future food-based dietary guidelines for Switzerland.

Our study has various strengths. The participants were drawn from national stratified random samples, and a weighting strategy was applied to the data, rendering the results representative for Switzerland. Additionally, since diet is one of the most important factors influencing health outcomes, the results of the present study might help to explain morbidity and mortality previously reported for Switzerland, pinpoint areas where dietary improvements are needed and highlight the need for frequent, high-quality assessment of the diet of people living in Switzerland.

The present study also has some limitations. Due to the lack of detailed dietary information in the SHS, we were not able to operationalise all food-based dietary guidelines included in the Swiss Food Pyramid. That can explain the differences observed between our study and a previous study using the menuCH data^(7)^. The differences in sampling methods and sizes of the study populations could have contributed to the varying results we detected between surveys. However, appropriate weights for both surveys were employed to account for these differences. Recall bias and the potential under- or over-reporting in the 24-h dietary recall interviews or the diet-related questions in the SHS cannot be excluded, as in any observational study relying on self-reported measures for dietary assessment. Study participants, including respondents of health surveys, are generally considered to be more health-conscious than the general population, suggesting that the proportions of individuals in the general population not meeting food-based dietary guidelines might be even higher.

In conclusion, a large proportion of the population in Switzerland was found to not meet the Swiss food-based dietary guidelines. Depending on the method of dietary assessment these proportions varied, highlighting differences in the diet based on the assessment method used. Education and policy actions are needed to raise awareness about the existence of national guidelines, remove barriers to healthy eating and make healthier foods the easiest and most readily accessible option to aid the population in adhering to the food-based dietary guidelines.

## Supplementary material

For supplementary material accompanying this paper visit https://doi.org/10.1017/S1368980022000647. click here to view supplementary material
