# Supplementary material for: Different dietary assessment methods, similar conclusions? Comparison of a country’s adherence to food-based dietary guidelines as depicted in two population-based surveys using different dietary assessment methods
Source: Public Health Nutr. 2022 Mar 21;25(9):2395–402. doi: 10.1017/S1368980022000647 (PMC9991603; doi:10.1017/S1368980022000647)
Supplement: Supplementary file 1 [file S1368980022000647sup.zip › S1368980022000647sup002.docx]

**STROBE-nut: An extension of the STROBE statement for nutritional epidemiology**

Lachat C et al. (2016) STrengthening the Reporting of OBservational studies in Epidemiology – Nutritional Epidemiology (STROBE-nut): an extension of the STROBE statement. Plos Medicine 13(6) <http://dx.doi.org/10.1371/journal.pmed.1002036> [pdf](http://journals.plos.org/plosmedicine/article/asset?id=10.1371%2Fjournal.pmed.1002036.PDF) or [online](http://journals.plos.org/plosmedicine/article?id=10.1371/journal.pmed.1002036) version.

**Different dietary assessment methods, similar conclusions? Comparison of a country’s diet depicted in two population-based surveys using different dietary assessment methods.**

| **Item** | **Item nr** | **STROBE recommendations** | **Extension for Nutritional Epidemiology studies (STROBE-nut)** | **Reported on page #** |
| --- | --- | --- | --- | --- |
| **Title and**  **abstract** | 1 | (a) Indicate the study’s design with a commonly used term in the title or the abstract.  The abstract indicates that these are cross-sectional surveys.  (b) Provide in the abstract an informative and balanced summary of what was done and what was found.  Yes. | **nut-1** State the dietary/nutritional assessment method(s) used in the title, abstract, or keywords.  24-hour dietary recalls and short dietary questionnaire are included in the abstract. | 1 |
| **Introduction** |  |  |  |  |
| Background/rationale | 2 | Explain the scientific background and rationale for the investigation being reported.  We explain the scientific background and reference to previous studies in the introduction. |  | 2 |
| Objectives | 3 | State specific objectives, including any pre-specified hypotheses.  We state the aim of the study in the last paragraph of the introduction. |  | 2 |
| **Methods** |  |  |  |  |
| Study design | 4 | Present key elements of study design early in the paper.  We give information about the survey designs in the first subsection of the methods section (“Data and study populations”). |  | 2-4 |
| Settings | 5 | Describe the setting, locations, and relevant dates, including periods of recruitment, exposure, follow-up, and data collection.  Information about recruitment and data collection is described in the first subsection of the methods section (“Data and study populations”). | **nut-5** Describe any characteristics of the study settings that might affect the dietary intake or nutritional status of the participants, if applicable.  Some considerations about dietary assessment are presented in the subsections “Data and study populations” and “Swiss food-based dietary guidelines and their operationalization in the datasets”. | 2-4 |
| Participants | 6 | a) Cohort study—Give the eligibility criteria, and the sources and methods of selection of participants. Describe methods of follow-up.  NA  Case-control study—Give the eligibility criteria, and the sources and methods of case ascertainment and control selection. Give the rationale for the choice of cases and controls.  NA  Cross-sectional study—Give the eligibility criteria, and the sources and methods of selection of participants.  Information is available in the methods subsection (“Data and study populations”).  (b) Cohort study—For matched studies, give matching criteria and number of exposed and unexposed.  NA  Case-control study—For matched studies, give matching criteria and the number of controls per case.  NA | **nut-6** Report particular dietary, physiological or nutritional characteristics that were considered when selecting the target population.  The populations were selected from a random stratified sample in order to be representative of the adult Swiss population. No particular dietary characteristics were therefore taken into account. | 2-4 |
| Variables | 7 | Clearly define all outcomes, exposures, predictors, potential confounders, and effect modifiers. Give diagnostic criteria, if applicable.  Classification of the dietary intake, operationalization of the food-based dietary guidelines and sociodemographic characteristics are given in the subsections “Sociodemographic and lifestyle variables” and “Swiss food-based dietary guidelines and their operationalization in the datasets”. | **nut-7.1** Clearly define foods, food groups, nutrients, or other food components.  Information about the food-based dietary guidelines were previously published. We referred to this (Swiss Society for Nutrition & Federal Food Safety and Veterinary Office. Swiss Food Pyramid, 2011).  **nut-7.2** When using dietary patterns or indices, describe the methods to obtain them and their nutritional properties.  NA | 5-9 |
| Data sources - measurements | 8 | For each variable of interest, give sources of data and details of methods of assessment (measurement). Describe comparability of assessment methods if there is more than one group.  The methods of assessment of the variables of interest are reported in the methods subsections describing the variables (“Data and study populations”). | **nut-8.1** Describe the dietary assessment method(s), e.g., portion size estimation, number of days and items recorded, how it was developed and administered, and how quality was assured. Report if and how supplement intake was assessed.  This information is presented in the methods subsections (“Data and study populations”, “Swiss food-based dietary guidelines and their operationalization in the datasets”).  **nut-8.2** Describe and justify food composition data used. Explain the procedure to match food composition with consumption data. Describe the use of conversion factors, if applicable.  NA  **nut-8.3** Describe the nutrient requirements, recommendations, or dietary guidelines and the evaluation approach used to compare intake with the dietary reference values, if applicable.  This information is presented in the methods subsection (“Swiss food-based dietary guidelines and their operationalization in the datasets”).  **nut-8.4** When using nutritional biomarkers, additionally use the STROBE Extension for Molecular Epidemiology (STROBE-ME). Report the type of biomarkers used and their usefulness as dietary exposure markers.  NA  **nut-8.5** Describe the assessment of non-dietary data (e.g., nutritional status and influencing factors) and timing of the assessment of these variables in relation to dietary assessment.  NA  **nut-8.6** Report on the validity of the dietary or nutritional assessment methods and any internal or external validation used in the study, if applicable.  Details are given in the Strengths and Limitations part of the discussion. | 2-4/9 |
| Bias | 9 | Describe any efforts to address potential sources of bias.  Information is given in the methods subsection “Statistical analysis”. Further details are presented in the Strengths und Limitations part of the discussion. | **nut-9** Report how bias in dietary or nutritional assessment was addressed, e.g., misreporting, changes in habits as a result of being measured, or data imputation from other sources  Dietary assessment data were cleaned and screened for inconsistency in the primary study (subsection “Data and study populations”). | 4-5/9 |
| Study Size | 10 | Explain how the study size was arrived at.  The study size depends on the national surveys. Details are given in the methods subsection (“Data and study populations”). |  | 2-4 |
| Quantitative variables | 11 | Explain how quantitative variables were handled in the analyses. If applicable, describe which groupings were chosen and why.  Details are given in the methods subsections “Sociodemographic and lifestyle variables” and “Swiss food-based dietary guidelines and their operationalization in the datasets”, as well as in Supplementary Table 1. | **nut-11** Explain categorization of dietary/nutritional data (e.g., use of N-tiles and handling of non-consumers) and the choice of reference category, if applicable.  Information is given in the methods subsection “Swiss food-based dietary guidelines and their operationalization in the datasets”. | 4 |
| Statistical  Methods | 12 | (a) Describe all statistical methods, including those used to control for confounding  Information is presented in the methods subsection “Statistical analysis”.  (b) Describe any methods used to examine subgroups and interactions.  Information is given in the methods subsection “Statistical analysis”.  (c) Explain how missing data were addressed.  Information about missing data is given in the methods subsection “Data and study populations”.  (d) Cohort study—If applicable, explain how loss to follow-up was addressed.  NA  Case-control study—If applicable, explain how matching of cases and controls was addressed.  NA  Cross-sectional study—If applicable, describe analytical methods taking account of sampling strategy.  We provide information about the weighting strategy applied in the surveys in the methods subsection “Statistical analysis” and in Tables 1- 3.  (e) Describe any sensitivity analyses.  We provide information about sensitivity analyses in the methods subsections in Table S2 of the supplementary materials. | **nut-12.1** Describe any statistical method used to combine dietary or nutritional data, if applicable.  NA  **nut-12.2** Describe and justify the method for energy adjustments, intake modeling, and use of weighting factors, if applicable.  Information is given in the methods subsection “Statistical analysis” and in Tables 1- 3.  **nut-12.3** Report any adjustments for measurement error, i.e,. from a validity or calibration study.  NA | 2-5 |
| **Results** |  |  |  |  |
| Participants | 13 | (a) Report the numbers of individuals at each stage of the study—e.g., numbers potentially eligible, examined for eligibility, confirmed eligible, included in the study, completing follow-up, and analyzed.  Information is given in the methods subsection “Data and study populations” and in Table 1.  (b) Give reasons for non-participation at each stage.  Information is given in the methods subsection “Data and study populations”.  (c) Consider use of a flow diagram.  NA | **nut-13** Report the number of individuals excluded based on missing, incomplete or implausible dietary/nutritional data.  We included all participants 18-75 years (see methods subsection “Data and study populations”). | 2-5 |
| Descriptive data | 14 | (a) Give characteristics of study participants (e.g., demographic, clinical, social) and information on exposures and potential confounders  Details are given in the results section and in Table 1 and Table 2.  (b) Indicate the number of participants with missing data for each variable of interest  Information is given in Table 1 and Table 2.  (c) Cohort study—Summarize follow-up time (e.g., average and total amount)  NA | **nut-14** Give the distribution of participant characteristics across the exposure variables if applicable. Specify if food consumption of total population or consumers only were used to obtain results.  The participant characteristics are presented in the results section and in Table 1 and Table 2. | 5-6 |
| Outcome data | 15 | Cohort study—Report numbers of outcome events or summary measures over time.  NA  Case-control study—Report numbers in each exposure category, or summary measures of exposure.  NA  Cross-sectional study—Report numbers of outcome events or summary measures.  NA |  |  |
| Main results | 16 | (a) Give unadjusted estimates and, if applicable, confounder-adjusted estimates and their precision (e.g., 95% confidence interval).  Make clear which confounders were adjusted for and why they were included.  NA  (b) Report category boundaries when continuous variables were categorized.  Categorization of variables is given in Tables 1-3.  (c) If relevant, consider translating estimates of relative risk into absolute risk for a meaningful time period.  NA | **nut-16** Specify if nutrient intakes are reported with or without inclusion of dietary supplement intake, if applicable.  NA | 4 |
| Other analyses | 17 | Report other analyses done—e.g., analyses of subgroups and interactions and sensitivity analyses.  We report additional analyses (see methods subsections “Statistical analysis”, results section and Table S2 of the supplementary materials). | **nut-17** Report any sensitivity analysis (e.g., exclusion of misreporters or outliers) and data imputation, if applicable.  We report additional analyses (see methods subsections “Statistical analysis”, results section and Table S2 of the supplementary materials). | 5 |
| **Discussion** |  |  |  |  |
| Key results | 18 | Summarize key results with reference to study objectives.  Information is given in the first paragraph of the discussion section. |  | 6 |
| Limitation | 19 | Discuss limitations of the study, taking into account sources of potential bias or imprecision. Discuss both direction and magnitude of any potential bias.  Information is given in the second to last paragraph of the discussion section. | **nut-19** Describe the main limitations of the data sources and assessment methods used and implications for the interpretation of the findings.  Information is given in the second to last paragraph of the discussion section. | 9 |
| Interpretation | 20 | Give a cautious overall interpretation of results considering objectives, limitations, multiplicity of analyses, results from similar studies, and other relevant evidence.  Interpretation of results and comparison to other studies is presented in the central part of the discussion. | **nut-20** Report the nutritional relevance of the findings, given the complexity of diet or nutrition as an exposure.  Information is given in the central part of the discussion and in the conclusion part. | 6-9 |
| Generalizability | 21 | Discuss the generalizability (external validity) of the study results.  Information is given in the Strengths and Limitations paragraphs of the discussion. |  | 9 |
| **Other information** |  |  |  |  |
| Funding | 22 | Give the source of funding and the role of the funders for the present study and, if applicable, for the original study on which the present article is based.  Information about the source of funding is given in the title page. |  |  |
| *Ethics* |  |  | **nut-22.1** Describe the procedure for consent and study approval from ethics committee(s).  Details are described in the title page subsection “Ethical standards disclosure”. |  |
| *Supplementary material* |  |  | **nut-22.2** Provide data collection tools and data as online material or explain how they can be accessed.  Information and references are given in the title page. |  |
