# Supplementary material for: Different dietary assessment methods, similar conclusions? Comparison of a country’s adherence to food-based dietary guidelines as depicted in two population-based surveys using different dietary assessment methods
Source: Public Health Nutr. 2022 Mar 21;25(9):2395–402. doi: 10.1017/S1368980022000647 (PMC9991603; doi:10.1017/S1368980022000647)
Supplement: Supplementary file 1 [file S1368980022000647sup.zip › S1368980022000647sup003.docx]

Supplementary Table 1. Operationalization of the Swiss food-based dietary guidelines in the two study populations

|  | Swiss Health Survey | menuCH survey |
| --- | --- | --- |
| Fruits | ≥ 2 portions/day or ≥ 14 portions/week, based on the SHS^1^ variables TERNA22 and TERNA23 | ≥ 2 portions as habitual food intake* |
| Vegetables | ≥ 3 portions/day or ≥ 21 portions/week, based on the SHS variables TERNA20 and TERNA21 | ≥ 3 portions as habitual food intake |
| Dairy products | ≥ 3 portions/day or ≥ 21 portions/week, based on the SHS variables TERNA18 and TERNA24 | ≥ 3 portions as habitual food intake |
| Meat and meat products | ≤ 3 times/week, based on the SHS variable TERNA03 | ≤ 35 g as habitual food intake |
| Fish | ≥ 1 times/week, based on the SHS variable TERNA05 | ≥ 20 g as habitual food intake |
| Alcohol | ≤ 1-2 times/week, based on the SHS variable TALKO15 | ≤ 7.5 g (for women) or 15 g (for men) as habitual intake |

^1^ SHS: Swiss Health Survey * Habitual intake was estimated using the Multiple Source Method.

Supplementary Table 2. Unweighted proportions of participants meeting the Swiss food-based dietary guidelines

|  | Swiss Health Survey, participants aged 18-75 (n=18,991) | menuCH survey, participants aged 18-75 (n=2,057) |
| --- | --- | --- |
| Fruits **(%)** |  |  |
| Meet | 37.6 | 37.8 |
| Do not meet | 62.3 | 62.2 |
| Missing | 0.1 | - |
| Vegetables **(%)** |  |  |
| Meet | 19.0 | 2.8 |
| Do not meet | 80.9 | 97.2 |
| Missing | 0.1 | - |
| Dairy products **(%)** |  |  |
| Meet | 8.9 | 13.1 |
| Do not meet | 91.0 | 86.9 |
| Missing | 0.1 | - |
| Meat and meat products **(%)** |  |  |
| Meet | 44.0 | 8.7 |
| Do not meet | 55.5 | 91.3 |
| Missing | 0.1 | - |
| Fish **(%)** |  |  |
| Meet | 66.1 | 31.0 |
| Do not meet | 33.8 | 69.0 |
| Missing | 0.1 | - |
| Alcohol **(%)** |  |  |
| Meet | 74.4 | 57.4 |
| Do not meet | 25.6 | 42.6 |
| Missing | 0.1 | - |

Supplementary Table 3. Proportions of participants meeting the Swiss food-based dietary guidelines when using non-adjusted vs. sex and age-adjusted models in the Multiple Source Method ^1^

|  | Non-adjusted | Sex and age-adjusted |
| --- | --- | --- |
| Fruits **(%)** | 37.9 | 37.8 |
| Vegetables **(%)** | 2.8 | 2.7 |
| Dairy products **(%)** | 13.5 | 13.2 |
| Meat and meat products **(%)** | 8.7 | 7.5 |
| Fish **(%)** | 32.5 | 32.7 |
| Alcohol **(%)** | 56.3 | 56.5 |

^1^ Survey weights used corrected for non-response based on six socio-demographic parameters (i.e., age, sex, marital status, major area, nationality, and household size) and uneven distribution of the two 24-hour dietary recall interviews over seasons and weekdays.
