# Supplementary material for: Different dietary assessment methods, similar conclusions? Comparison of a country’s adherence to food-based dietary guidelines as depicted in two population-based surveys using different dietary assessment methods
Source: Public Health Nutr. 2022 Mar 21;25(9):2395–402. doi: 10.1017/S1368980022000647 (PMC9991603; doi:10.1017/S1368980022000647)
Supplement: Supplementary file 1 [file S1368980022000647sup.zip › S1368980022000647sup001.doc]

**SUPPLEMENTARY MATERIAL**

**Table 1 –** Characterization of the food groups promoted on an online food delivery platform during the COVID-19 pandemic in Belo Horizonte, during lunch**.**

**Table 2 –** Characterization of the food groups promoted on an online food delivery platform during the COVID-19 pandemic in Belo Horizonte, during dinner**.**

**Table 3 –** Characterization of the food groups promoted on an online food delivery platform during the COVID-19 pandemic in Belo Horizonte, during weekday**.**

**Table 4 –** Characterization of the food groups promoted on an online food delivery platform during the COVID-19 pandemic in Belo Horizonte, during weekend**.**

**Table 1 – Characterization of the food groups promoted on an online food delivery platform during the COVID-19 pandemic in Belo Horizonte, during lunch**.

| **Variable** | **Week** | | | | | | | | | | | | | | | |
| --- | --- | --- | --- | --- | --- | --- | --- | --- | --- | --- | --- | --- | --- | --- | --- | --- |
|  | **1** | **2** | **3** | **4** | **5** | **6** | **7** | **8** | **9** | **10** | **11** | **12** | **13** | **14** | **15** | **16** |
| **Food groups** |  |  |  |  |  |  |  |  |  |  |  |  |  |  |  |  |
| Water (%) | 0.0 | 0.0 | 0.0 | 0.0 | 0.0 | 0.0 | 0.0 | 0.0 | 0.0 | 0.0 | 0.0 | 0.0 | 0.0 | 0.0 | 0.0 | 0.0 |
| Natural juices and smoothies (%) | 1.9 | 2.0 | 4.1 | 6.1 | 0.0 | 6.0 | 4.1 | 4.0 | 2.0 | 8.0 | 2.0 | 2.0 | 8.0 | 4.1 | 4.0 | 4.3 |
| Vegetables (%) | 1.9 | 2.0 | 4.1 | 0.0 | 4.0 | 4.0 | 6.1 | 4.0 | 4.0 | 10.0 | 10.0 | 2.0 | 8.0 | 10.2 | 6.0 | 2.1 |
| Fruits (%) | 1.9 | 0.0 | 0.0 | 0.0 | 0.0 | 0.0 | 0.0 | 0.0 | 0.0 | 0.0 | 0.0 | 2.0 | 2.0 | 0.0 | 0.0 | 0.0 |
| Traditional meals or pasta (%) | 28.9 | 30.0 | 36.7 | 44.9 | 34.0 | 42.0 | 46.9 | 24.0 | 50.0 | 44.0 | 38.0 | 36.0 | 44.0 | 40.8 | 34.0 | 42.6 |
| Ultra-processed beverages (%) | 15.4 | 12.0 | 16.3 | 16.3 | 18.0 | 14.0 | 8.2 | 22.0 | 6.0 | 8.0 | 18.0 | 24.0 | 14.0 | 18.4 | 22.0 | 25.5 |
| Ice-cream, candies, or salty packaged snacks (%) | 7.7 | 10.0 | 8.2 | 10.2 | 10.0 | 6.0 | 2.0 | 6.0 | 8.0 | 4.0 | 8.0 | 4.0 | 6.0 | 0.0 | 4.0 | 6.4 |
| Sandwiches (%) | 15.4 | 16.0 | 12.2 | 10.2 | 8.0 | 10.0 | 6.1 | 30.0 | 10.0 | 6.0 | 12.0 | 16.0 | 8.0 | 20.4 | 18.0 | 12.8 |
| Savoury snacks (%) | 0.0 | 2.0 | 10.2 | 2.0 | 2.0 | 4.0 | 6.1 | 4.0 | 0.0 | 2.0 | 2.0 | 4.0 | 12.0 | 6.1 | 8.0 | 8.5 |
| Pizza (%) | 9.6 | 0.0 | 8.2 | 4.1 | 10.0 | 0.0 | 4.1 | 6.0 | 10.0 | 10.0 | 14.0 | 12.0 | 4.0 | 8.2 | 10.0 | 12.8 |
| **Marketing strategies** |  |  |  |  |  |  |  |  |  |  |  |  |  |  |  |  |
| Combos (%) | 23.1 | 18.0 | 24.5 | 22.5 | 16.0 | 18.0 | 16.3 | 34.0 | 14.0 | 18.0 | 30.0 | 28.0 | 30.0 | 32.7 | 38.0 | 36.2 |
| Photos (%) | 84.6 | 72.0 | 77.6 | 89.8 | 82.0 | 94.0 | 83.7 | 86.0 | 94.0 | 94.0 | 98.0 | 92.0 | 98.0 | 87.8 | 98.0 | 89.4 |
| Discounts (%) | 100.0 | 100.0 | 97.9 | 100.0 | 100.0 | 96.0 | 97.9 | 100.0 | 100.0 | 98.0 | 100.0 | 96.0 | 100.0 | 100.0 | 98.0 | 97.8 |
| Message on healthiness (%) | 1.9 | 0.0 | 4.1 | 0.0 | 0.0 | 14.0 | 4.1 | 2.0 | 6.0 | 6.0 | 6.0 | 8.0 | 8.0 | 8.2 | 8.0 | 2.1 |
| Message on value for the money (%) | 17.3 | 20.0 | 24.5 | 28.6 | 22.0 | 22.0 | 22.5 | 24.0 | 26.0 | 30.0 | 26.0 | 18.0 | 16.0 | 18.4 | 24.0 | 19.2 |
| Message of tastiness and pleasure (%) | 23.1 | 14.0 | 34.7 | 32.7 | 22.0 | 32.0 | 24.5 | 20.0 | 12.0 | 22.0 | 26.0 | 20.0 | 18.0 | 18.4 | 32.0 | 21.3 |

**Table 2 – Characterization of the food groups promoted on an online food delivery platform during the COVID-19 pandemic in Belo Horizonte, during dinner**.

| **Variable** | **Week** | | | | | | | | | | | | | | | |
| --- | --- | --- | --- | --- | --- | --- | --- | --- | --- | --- | --- | --- | --- | --- | --- | --- |
|  | **1** | **2** | **3** | **4** | **5** | **6** | **7** | **8** | **9** | **10** | **11** | **12** | **13** | **14** | **15** | **16** |
| **Food groups** |  |  |  |  |  |  |  |  |  |  |  |  |  |  |  |  |
| Water (%) | 0.0 | 0.0 | 0.0 | 0.0 | 0.0 | 0.0 | 0.0 | 0.0 | 0.0 | 0.0 | 0.0 | 0.0 | 0.0 | 0.0 | 0.0 | 0.0 |
| Natural juices and smoothies (%) | 3.9 | 3.9 | 3.9 | 7.6 | 0.0 | 7.6 | 7.7 | 11.5 | 2.0 | 11.1 | 3.9 | 1.9 | 7.4 | 3.9 | 5.8 | 6.1 |
| Vegetables (%) | 2.0 | 2.0 | 2.0 | 4.0 | 2.0 | 2.0 | 4.0 | 0.0 | 6.1 | 6.0 | 0.0 | 1.9 | 4.0 | 4.0 | 6.0 | 4.3 |
| Fruits (%) | 2.0 | 0.0 | 0.0 | 0.0 | 2.0 | 2.0 | 0.0 | 0.0 | 0.0 | 0.0 | 0.0 | 0.0 | 0.0 | 0.0 | 0.0 | 0.0 |
| Traditional meals or pasta (%) | 6.0 | 14.0 | 8.0 | 10.0 | 10.0 | 18.0 | 14.0 | 14.0 | 12.2 | 12.0 | 18.0 | 13.5 | 18.0 | 16.0 | 12.0 | 17.0 |
| Ultra-processed beverages (%) | 26.0 | 32.0 | 26.0 | 42.0 | 28.0 | 32.0 | 24.0 | 32.0 | 18.4 | 26.0 | 20.0 | 26.9 | 26.0 | 30.0 | 22.0 | 23.4 |
| Ice-cream, candies, or salty packaged snacks (%) | 4.0 | 8.0 | 8.0 | 8.0 | 6.0 | 6.0 | 8.0 | 4.0 | 8.2 | 8.0 | 12.0 | 7.7 | 8.0 | 6.0 | 12.0 | 6.4 |
| Sandwiches (%) | 26.0 | 34.0 | 32.0 | 38.0 | 24.0 | 28.0 | 18.0 | 26.0 | 32.7 | 28.0 | 28.0 | 25.0 | 34.0 | 30.0 | 40.0 | 25.5 |
| Savoury snacks (%) | 4.0 | 2.0 | 2.0 | 0.0 | 2.0 | 2.0 | 10.0 | 8.0 | 6.1 | 6.0 | 4.0 | 1.9 | 4.0 | 2.0 | 6.0 | 2.1 |
| Pizzas (%) | 24.0 | 16.0 | 14.0 | 12.0 | 26.0 | 20.0 | 24.0 | 18.0 | 12.2 | 16.0 | 4.0 | 25.0 | 16.0 | 10.0 | 12.0 | 19.2 |
| **Marketing strategies** |  |  |  |  |  |  |  |  |  |  |  |  |  |  |  |  |
| Combos (%) | 32.0 | 38.0 | 38.0 | 46.0 | 36.0 | 34.0 | 34.0 | 42.0 | 24.5 | 28.0 | 30.0 | 30.8 | 44.0 | 42.0 | 46.0 | 29.8 |
| Photos (%) | 86.0 | 84.0 | 84.0 | 76.0 | 80.0 | 80.0 | 86.0 | 92.0 | 95.9 | 100.0 | 94.0 | 86.5 | 94.0 | 100.0 | 98.0 | 83.0 |
| Discounts (%) | 100.0 | 100.0 | 98.0 | 100.0 | 100.0 | 98.0 | 100.0 | 100.0 | 100.0 | 100.0 | 100.0 | 100.0 | 100.0 | 100.0 | 96.0 | 100.0 |
| Message on healthiness (%) | 2.0 | 0.0 | 0.0 | 2.0 | 0.0 | 2.0 | 0.0 | 0.0 | 2.0 | 6.0 | 8.0 | 1.9 | 2.0 | 4.0 | 2.0 | 0.0 |
| Message on value for the money (%) | 22.0 | 22.0 | 22.0 | 14.0 | 20.0 | 20.0 | 22.0 | 26.0 | 38.8 | 30.0 | 22.0 | 21.2 | 36.0 | 18.0 | 44.0 | 19.2 |
| Message of tastiness and pleasure (%) | 16.0 | 26.0 | 42.0 | 26.0 | 14.0 | 24.0 | 26.0 | 10.0 | 20.4 | 18.0 | 30.0 | 17.3 | 28.0 | 28.0 | 28.0 | 34.0 |

**Table 3 – Characterization of the food groups promoted on an online food delivery platform during the COVID-19 pandemic in Belo Horizonte, during weekday**.

| **Variable** | **Week** | | | | | | | | | | | | | | | |
| --- | --- | --- | --- | --- | --- | --- | --- | --- | --- | --- | --- | --- | --- | --- | --- | --- |
|  | **1** | **2** | **3** | **4** | **5** | **6** | **7** | **8** | **9** | **10** | **11** | **12** | **13** | **14** | **15** | **16** |
| **Food groups** |  |  |  |  |  |  |  |  |  |  |  |  |  |  |  |  |
| Water (%) | 0.0 | 0.0 | 0.0 | 0.0 | 0.0 | 0.0 | 0.0 | 0.0 | 0.0 | 0.0 | 0.0 | 0.0 | 0.0 | 0.0 | 0.0 | 0.0 |
| Natural juices and smoothies (%) | 1.9 | 0.0 | 2.0 | 6.1 | 0.0 | 2.0 | 2.0 | 10.0 | 2.0 | 10.0 | 4.0 | 0.0 | 6.0 | 4.1 | 4.0 | 0.0 |
| Vegetables (%) | 0.0 | 2.0 | 4.1 | 2.0 | 2.0 | 6.0 | 6.0 | 0.0 | 10.0 | 4.0 | 6.0 | 2.0 | 8.0 | 6.1 | 12.0 | 6.0 |
| Fruits (%) | 0.0 | 0.0 | 0.0 | 0.0 | 0.0 | 2.0 | 0.0 | 0.0 | 0.0 | 0.0 | 0.0 | 0.0 | 0.0 | 0.0 | 0.0 | 0.0 |
| Traditional meals or pasta (%) | 21.2 | 28.0 | 24.5 | 26.5 | 22.0 | 30.0 | 40.0 | 14.0 | 28.0 | 30.0 | 28.0 | 21.6 | 28.0 | 32.7 | 18.0 | 34.0 |
| Ultra-processed beverages (%) | 13.5 | 20.0 | 16.3 | 32.7 | 26.0 | 26.0 | 14.0 | 30.0 | 14.0 | 20.0 | 14.0 | 25.5 | 20.0 | 22.5 | 24.0 | 18.0 |
| Ice-cream, candies, or salty packaged snacks (%) | 5.8 | 4.0 | 10.2 | 8.2 | 6.0 | 4.0 | 2.0 | 6.0 | 6.0 | 4.0 | 12.0 | 7.8 | 4.0 | 0.0 | 6.0 | 12.0 |
| Sandwiches (%) | 17.3 | 22.0 | 12.2 | 26.5 | 20.0 | 18.0 | 10.0 | 28.0 | 24.0 | 18.0 | 18.0 | 27.5 | 22.0 | 28.6 | 30.0 | 24.0 |
| Savoury snacks (%) | 1.9 | 2.0 | 4.1 | 0.0 | 2.0 | 4.0 | 8.0 | 8.0 | 4.0 | 6.0 | 2.0 | 2.0 | 10.0 | 4.1 | 8.0 | 4.0 |
| Pizzas (%) | 17.3 | 6.0 | 12.2 | 10.2 | 22.0 | 8.0 | 18.0 | 12.0 | 10.0 | 10.0 | 6.0 | 21.6 | 8.0 | 8.2 | 12.0 | 8.0 |
| **Marketing strategies** |  |  |  |  |  |  |  |  |  |  |  |  |  |  |  |  |
| Combos (%) | 21.2 | 20.0 | 22.5 | 38.8 | 32.0 | 26.0 | 22.0 | 48.0 | 22.0 | 28.0 | 26.0 | 27.5 | 38.0 | 42.9 | 44.0 | 32.0 |
| Photos (%) | 84.6 | 70.0 | 75.5 | 79.6 | 76.0 | 96.0 | 76.0 | 90.0 | 94.0 | 98.0 | 96.0 | 82.4 | 92.0 | 89.8 | 100.0 | 90.0 |
| Discounts (%) | 100.0 | 100.0 | 95.9 | 100.0 | 100.0 | 94.0 | 98.0 | 100.0 | 100.0 | 100.0 | 100.0 | 98.0 | 100.0 | 100.0 | 98.0 | 100.0 |
| Message on healthiness (%) | 1.9 | 0.0 | 4.1 | 0.0 | 0.0 | 8.0 | 2.0 | 0.0 | 6.0 | 8.0 | 10.0 | 3.9 | 6.0 | 6.1 | 4.0 | 2.0 |
| Message on value for the money (%) | 21.2 | 22.0 | 26.5 | 18.4 | 16.0 | 22.0 | 18.0 | 30.0 | 34.0 | 28.0 | 32.0 | 17.7 | 16.0 | 24.5 | 34.0 | 20.0 |
| Message of tastiness and pleasure (%) | 17.3 | 20.0 | 36.7 | 30.6 | 16.0 | 24.0 | 26.0 | 26.0 | 16.0 | 20.0 | 26.0 | 15.7 | 22.0 | 20.4 | 28.0 | 40.0 |

**Table 4 – Characterization of the food groups promoted on an online food delivery platform during the COVID-19 pandemic in Belo Horizonte, during weekend**.

| **Variable** | **Week** | | | | | | | | | | | | | | | |
| --- | --- | --- | --- | --- | --- | --- | --- | --- | --- | --- | --- | --- | --- | --- | --- | --- |
|  | **1** | **2** | **3** | **4** | **5** | **6** | **7** | **8** | **9** | **10** | **11** | **12** | **13** | **14** | **15** | **16** |
| **Food groups** |  |  |  |  |  |  |  |  |  |  |  |  |  |  |  |  |
| Water (%) | 0.0 | 0.0 | 0.0 | 0.0 | 0.0 | 0.0 | 0.0 | 0.0 | 0.0 | 0.0 | 0.0 | 0.0 | 0.0 | 0.0 | 0.0 | 0.0 |
| Natural juices and smoothies (%) | 2.0 | 4.0 | 2.0 | 2.0 | 0.0 | 6.0 | 6.1 | 2.0 | 0.0 | 2.0 | 0.0 | 2.0 | 2.0 | 0.0 | 2.0 | 6.8 |
| Vegetables (%) | 4.0 | 2.0 | 2.0 | 2.0 | 4.0 | 0.0 | 4.1 | 4.0 | 0.0 | 12.0 | 4.0 | 2.0 | 4.0 | 8.0 | 0.0 | 0.0 |
| Fruits (%) | 2.0 | 0.0 | 0.0 | 0.0 | 2.0 | 0.0 | 0.0 | 0.0 | 0.0 | 0.0 | 0.0 | 2.0 | 2.0 | 0.0 | 0.0 | 0.0 |
| Traditional meals or pasta (%) | 14.0 | 16.0 | 20.0 | 28.0 | 22.0 | 30.0 | 20.4 | 24.0 | 34.7 | 26.0 | 28.0 | 27.5 | 34.0 | 24.0 | 28.0 | 25.0 |
| Ultra-processed beverages (%) | 28.0 | 24.0 | 26.0 | 26.0 | 20.0 | 20.0 | 18.4 | 24.0 | 10.2 | 14.0 | 24.0 | 25.5 | 20.0 | 26.0 | 20.0 | 31.8 |
| Ice-cream, candies, or salty packaged snacks (%) | 6.0 | 14.0 | 6.0 | 10.0 | 10.0 | 8.0 | 8.2 | 4.0 | 10.2 | 8.0 | 8.0 | 3.9 | 10.0 | 6.0 | 10.0 | 0.0 |
| Sandwiches (%) | 24.0 | 28.0 | 32.0 | 22.0 | 12.0 | 20.0 | 14.3 | 28.0 | 18.4 | 16.0 | 22.0 | 13.7 | 20.0 | 22.0 | 28.0 | 13.6 |
| Savoury snacks (%) | 2.0 | 2.0 | 8.0 | 2.0 | 2.0 | 2.0 | 8.2 | 4.0 | 2.0 | 2.0 | 4.0 | 3.9 | 6.0 | 4.0 | 6.0 | 6.8 |
| Pizzas (%) | 16.0 | 10.0 | 10.0 | 6.0 | 14.0 | 12.0 | 10.2 | 12.0 | 12.2 | 16.0 | 12.0 | 15.7 | 12.0 | 10.0 | 10.0 | 25.0 |
| **Marketing strategies** |  |  |  |  |  |  |  |  |  |  |  |  |  |  |  |  |
| Combos (%) | 34.0 | 36.0 | 40.0 | 30.0 | 20.0 | 26.0 | 28.6 | 28.0 | 16.3 | 18.0 | 34.0 | 31.4 | 36.0 | 32.0 | 40.0 | 34.1 |
| Photos (%) | 86.0 | 86.0 | 86.0 | 86.0 | 86.0 | 78.0 | 93.9 | 88.0 | 95.9 | 96.0 | 96.0 | 96.1 | 100.0 | 98.0 | 96.0 | 81.8 |
| Discounts (%) | 100.0 | 100.0 | 100.0 | 100.0 | 100.0 | 100.0 | 100.0 | 100.0 | 100.0 | 98.0 | 100.0 | 98.0 | 100.0 | 100.0 | 96.0 | 97.7 |
| Message on healthiness (%) | 2.0 | 0.0 | 0.0 | 2.0 | 0.0 | 8.0 | 2.0 | 2.0 | 2.0 | 4.0 | 4.0 | 5.9 | 4.0 | 6.0 | 6.0 | 0.0 |
| Message on value for the money (%) | 18.0 | 20.0 | 20.0 | 24.0 | 26.0 | 20.0 | 26.5 | 20.0 | 30.6 | 32.0 | 16.0 | 21.6 | 36.0 | 12.0 | 34.0 | 18.2 |
| Message of tastiness and pleasure (%) | 22.0 | 20.0 | 40.0 | 28.0 | 20.0 | 32.0 | 24.5 | 4.0 | 16.3 | 20.0 | 30.0 | 21.6 | 24.0 | 26.0 | 32.0 | 13.6 |
